# Asymptomatic Uterine Rupture in a Pregnancy Following B‐Lynch Suture Application in a Previous Delivery: A Case Report

**DOI:** 10.1002/ccr3.71053

**Published:** 2025-09-29

**Authors:** Kareem Omran, Gyia Mathew, Maha Shahin, Osama Omran

**Affiliations:** ^1^ Faculty of Life Sciences & Medicine King's College London London UK; ^2^ Obstetrics & Gynaecology Al Qassimi Women's and Children's Hospital Sharjah UAE; ^3^ Obstetrics & Gynaecology Medcare Hospital Sharjah Sharjah UAE

**Keywords:** B‐Lynch suture, polydioxanone, postpartum hemorrhage, pregnancy complications, uterine compression suture, uterine rupture

## Abstract

The B‐Lynch suture, designed to control severe postpartum hemorrhage by compressing the uterus, is effective in reducing maternal mortality rates. However, it can lead to complications such as infection and uterine necrosis. This report presents a rare case of asymptomatic uterine rupture in a subsequent pregnancy following a B‐Lynch suture. A woman in her mid‐30s, gravida 3, para 2, presented at 32 weeks gestation in labor and underwent a repeat caesarean section. During delivery and visualization of the uterus, a 3‐cm uterine rent was incidentally discovered near the site of the previous B‐Lynch suture at the posterior aspect of the uterus. On review of prior medical reports provided by the patient, it was noted that the B‐Lynch procedure was previously performed using Polydioxanone sutures rather than traditional absorbable sutures and was deemed a potential contributor to this presentation. This case emphasizes the importance of appropriate suture materials and techniques when performing a B‐Lynch suture and presents a less commonly known potential long‐term effect and complication. Further, obstetricians should be wary in subsequent pregnancies of the potential for uterine rents and aim to carefully visualize the previous suture site intraoperatively, regardless of symptomatology.

**Summary**

- Asymptomatic uterine rupture in subsequent pregnancies is a potential complication of B‐Lynch sutures.
- Adherence to the use of appropriate suture materials and technique is crucial to prevent this long‐term complication.
- Awareness of this complication and early identification intraoperatively through careful visualization of the uterus is critical for ensuring patient safety.

## 1 Background

The B‐Lynch suture is a significant advancement in the field of obstetrics, particularly in the management of postpartum hemorrhage (PPH), which is a leading cause of maternal mortality worldwide [1]. Excessive bleeding after childbirth can quickly become life‐threatening if not addressed promptly and effectively. The B‐Lynch suture technique, named after Dr. Christopher B‐Lynch, who developed it in the late 20th century, is a form of brace suture applied to a uterus that is not contracting adequately after delivery [2]. It is designed to mechanically compress the uterine muscle, facilitating hemostasis without the need for an emergency peripartum hysterectomy. This procedure is especially valuable as it allows for the preservation of the uterus, thereby offering women the possibility of future pregnancies. Furthermore, the simplicity and cost‐effectiveness of the technique make it accessible even in low‐resource settings, playing a crucial role in reducing maternal mortality rates across the globe [2]. Although uterine necrosis is recognized as a potential complication post B‐Lynch suture application, there is a paucity in the literature concerning the risk of asymptomatic spontaneous dehiscence or uterine rupture in subsequent pregnancies.

## 2 Case History

A woman in her mid‐30s (Gravida 3, Para 2) presented to the Emergency Department at 32 weeks of gestation with complaints of lower abdominal pain consistent with labor contractions. She reported that the symptoms had been progressively intensifying over the preceding hours. This pregnancy had been unbooked, with no antenatal visits or screening documented prior to presentation. Her obstetric history included a first pregnancy resulting in a caesarean section at 28 weeks' gestation due to preterm labor in a twin pregnancy following in vitro fertilization (IVF), and a second caesarean at 33 weeks performed at a different hospital, complicated by uterine atony and significant intrapartum hemorrhage. Hemostasis had been achieved using Polydioxanone (PDS) B‐Lynch compression sutures, avoiding the need for hysterectomy. The patient presented with medical records from the previous hospital, which documented the use of a “B‐Lynch suture” without further technical specification. Based on this documentation, it was assumed that a standard B‐Lynch technique had been performed rather than a modified B‐Lynch or Hayman suture variant. There were no known medical comorbidities or allergies. She was not on regular medications, and there was no history of trauma or recent illness during this pregnancy.

## 3 Investigations and Treatment

On arrival, the patient was alert but visibly distressed due to frequent, painful uterine contractions. Her vital signs were within normal limits, and there were no signs of haemodynamic compromise. Obstetric examination revealed a symphysiofundal height consistent with 32 weeks' gestation, with palpable regular contractions.

Foetal monitoring using cardiotocography (CTG) showed a reactive trace, and bedside ultrasound confirmed a single live foetus in cephalic presentation, with an estimated foetal weight appropriate for gestational age and normal amniotic fluid volume. Placental location was anterior and away from the os, and there were no signs of placental abruption. Given her previous obstetric history and signs of established preterm labor, the decision was made to proceed with an emergency lower segment caesarean section.

**FIGURE 1:**
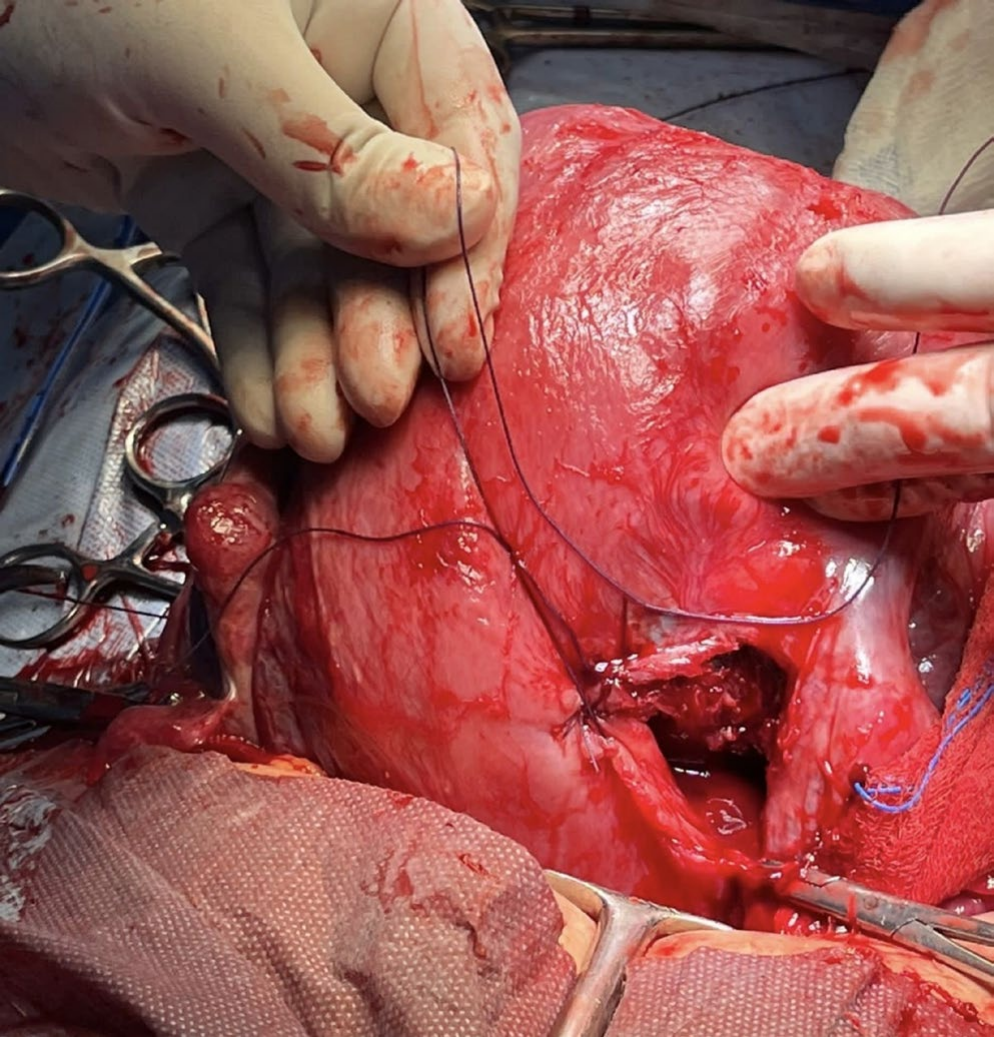
A 3‐cm rent on the posterior aspect of the uterus. The location corresponds to the site of the knot from the previously placed B‐Lynch suture.

A live male infant was delivered with a birth weight of 1.9 kg and Apgar scores of 8 and 9 at 1 and 5 min, respectively. The placenta was delivered with ease and was complete upon examination, with the placental bed located anteriorly away from the subsequently identified uterine defect. Following standard closure of the uterine incision, a 3‐cm rent was unexpectedly identified on the posterior uterine wall, close to the site of the previous B‐Lynch sutures (Figure 1). The tear did not extend into the broad ligament or cervix.

**FIGURE 2:**
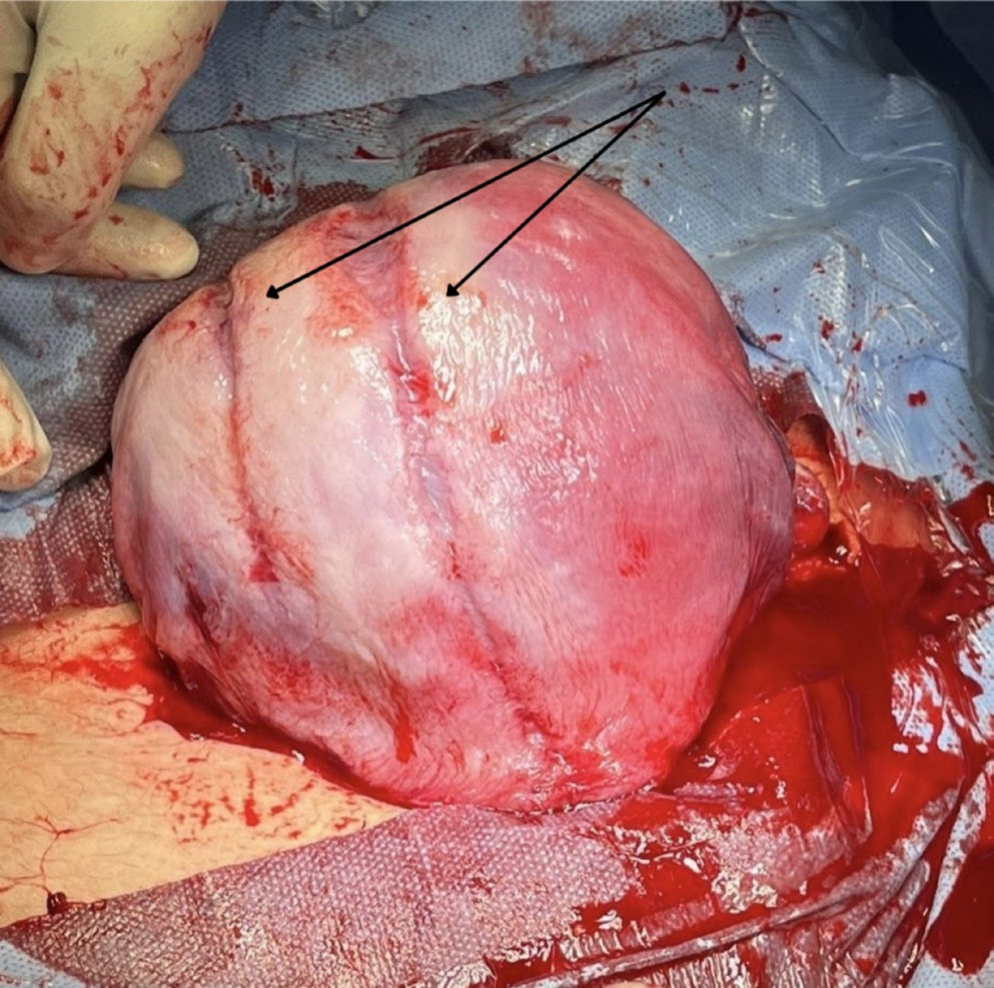
A close‐up view of the uterus with visible indentation marks on the fundal region, indicative of the placement of previous B‐Lynch sutures.

The rent was repaired in two layers using absorbable sutures, achieving complete hemostasis. On inspection, several indentation marks were noted on the posterior uterine wall, consistent with prior B‐Lynch suture placement (Figure 2). No active bleeding or extension of the defect was observed. There was no observable or reasonable cause for the posterior rent except for the presence of the previous compression suture, which likely led to pressure ischaemia of the uterine wall. The defect was located away from the placental bed, making placental causes such as abnormal adherence unlikely contributors. Prophylactic uterotonics were administered intraoperatively, and the uterus contracted well. No additional surgical intervention was required. Estimated blood loss was within expected limits and did not necessitate transfusion.

## 4 Outcome and Follow‐Up

Postoperatively, the patient was monitored in the maternity high‐dependency unit and made an uneventful recovery. Her hemoglobin levels remained stable, and there were no signs of infection or delayed hemorrhage. She was discharged home on postoperative day 4 with standard postnatal care instructions. A detailed plan for contraception and future pregnancy counseling was documented, including discussion of the risks associated with repeat caesarean sections and uterine rupture. The infant was admitted briefly to the neonatal unit for observation due to prematurity but did not require respiratory support and was subsequently discharged in good health.

At her 6‐week outpatient follow‐up, the patient reported no concerns. Wound healing was complete, and she remained clinically well. Repeat pelvic ultrasound showed no intrauterine collections or structural abnormalities. She was advised to undergo close surveillance in any future pregnancies and referred for preconception counseling.

## 5 Discussion

Atonic uterus is the predominant cause of postpartum hemorrhage and is managed in a conservative step‐wise approach [3]. The World Health Organization (WHO) recommends the application of prophylactic uterotonics for PPH in all births, with oxytocin as the cornerstone in initial treatment efforts [4]. Alternative conservative strategies, including balloon tamponade, selective ligation, and arterial embolization, play a crucial role in non‐surgical management when uterotonics are unsuccessful [5, 6]. However, the introduction of the B‐Lynch suture and its variants has been a game‐changer, offering a conservative surgical option aimed at preserving the uterus. Developed in 1997, this technique has shown an impressive 94% efficacy in controlling PPH, leading to its widespread acceptance [7, 8]. Initially, this technique was described through the use of chromic catgut sutures, and in a later report, No. 1 poliglecaprone‐25 suture, both of which are absorbable sutures [9].

The B‐Lynch suture technique is considered relatively safe due to having no risk of ureteric or major vessel injury, as well as its unique approach of not stitching the anterior and posterior walls of the uterus together, a method distinct from other compression sutures [2, 8]. In contrast, certain uterine compression sutures that do stitch these walls together are associated with a higher incidence of complications, including pyometra, hematometra, infection, ischemic necrosis, Asherman syndrome, and infertility [8]. However, despite the success of the B‐Lynch suture, the technique is associated with a range of complications, including myometrial damage, uterine rupture, adhesion formation, menstrual irregularities, and potential infertility [10, 11, 12]. The challenge lies in the pre‐delivery recognition of such risks.

Given the potential long‐term complications associated with uterine compression sutures, it is essential that operative records clearly document the specific technique used and the type of suture material applied during the management of postpartum hemorrhage. This is particularly important following B‐Lynch suture placement, as there is a documented risk of uterine rupture in subsequent pregnancies—most notably at the site of suture application, where the uterine wall may have been weakened [13]. Uterine rupture during late pregnancy or labor can pose life‐threatening risks to both the mother and fetus. The extent of this risk is influenced by factors such as the surgical method employed and the tensile properties of the suture material. In the present case, the use of non‐absorbable sutures—an unorthodox choice—may have contributed to excessive compression, thereby increasing the likelihood of ischemic pressure necrosis [8]. Polydioxanone (PDS), in particular, is known for its prolonged retention of tensile strength and slow absorption profile, which, if tied too tightly, may exacerbate tissue injury [14]. This case highlights the need to revisit established protocols and adhere to the original recommendations for B‐Lynch suture application, including the use of absorbable materials and appropriate tensioning techniques.

Furthermore, intraoperatively, during caesarean sections following a previous B‐Lynch suture, a thorough examination of the uterus is imperative. Both the anterior and posterior aspects of the uterus should be scrutinized for any signs of silent defects or weak points that could jeopardize future pregnancies. This approach is critical for identifying and addressing issues that, if left unattended, could lead to severe complications in the future.

Moreover, this case brings forth the broader implications of surgical interventions in obstetrics. The balance between immediate management of PPH and the long‐term health of the uterus is delicate. Every surgical decision, including suture choice and technique, can have profound implications on future fertility and pregnancy outcomes [15]. As such, continuous education and training in the latest techniques and materials are essential for obstetricians [16]. Patients with a history of PPH managed by techniques like the B‐Lynch suture should be counseled about the potential risks in future pregnancies. This information is crucial for informed decision‐making and planning, particularly at the time of the next delivery.

This case also highlights the challenges faced when managing patients who have undergone previous procedures at different institutions. The limited operative documentation available from the index surgery prevented detailed analysis of the exact surgical technique and concurrent interventions employed. Complete operative records, including specific suture materials, tensioning techniques, and timing of suture placement relative to uterine closure, are essential for optimal management of subsequent pregnancies.

## Author Contributions

**Kareem Omran:** conceptualization, data curation, formal analysis, investigation, methodology, validation, visualization, writing – original draft, writing – review and editing. **Gyia Mathew:** investigation, methodology, supervision, visualization, writing – original draft, writing – review and editing. **Maha Shahin:** conceptualization, formal analysis, investigation, supervision, validation, visualization, writing – original draft, writing – review and editing. **Osama Omran:** conceptualization, data curation, formal analysis, investigation, methodology, project administration, supervision, validation, visualization, writing – original draft, writing – review and editing.

## Consent

Written informed consent was obtained from the patient to publish this report in accordance with the journal's patient consent policy.

## Data Availability

No datasets were generated or analyzed during the current study. All relevant clinical information is included within the article.
